# An observational field study of the barriers preventing successful treatment, control and prevention of malaria in the forest tribal area of Rampachodavaram in Andhra Pradesh, India

**DOI:** 10.1186/1475-2875-11-S1-P54

**Published:** 2012-10-15

**Authors:** Lakshmi Karuparthy, Aparna Dasaraju, Srividya Dasaraju, Rekha Karuparthy, Satyadev Guthula, Rajini Dasaraju, Venkateswara Rao Karuparthy

**Affiliations:** 1 Pleasant Valley High School, Bettendorf, Iowa, 52722, USA; 2 Tufts University, Medford, Massachusetts, 02155, USA; 3 Washburn Rural High School, Topeka, Kansas, 66610, USA; 4 Pleasant Valley High School, Bettendorf, Iowa, 52722, USA; 5 Municipal Health Officer, Municipal Corporation Rajahmundry AP, 533104, India; 6 Washburn University, Topeka, Kansas, 66619, USA; 7 Vedic International Institute for Science and Arts, Bettendorf, Iowa, 52722, USA

## Background

Rampachodavaram and its surrounding areas, a densely forested tribal location, tout one of the highest incidence rates of malaria in Andhra Pradesh, India [1]. The rural health care network seems sufficient, however, the prevalence of malaria suggests deficiencies. Government statistics report zero deaths in the forested tribal areas from malaria [2]; yet, field observation proves the contrary. The situation calls for focus, awareness and attention. This preliminary study focuses on uncovering the challenges and barriers for effective malaria control.

## Materials and methods

We travelled into the interior forest area to speak with and observe villagers, physicians and health officials at individual huts, rural governmental/non-governmental health centers and offices respectively. Structured interviews were conducted with all three groups and the responses were analyzed side by side. In addition, we reviewed official reports and records of malaria statistics to gain a holistic understanding of the procedures and policies regarding malaria control in the tribal area.

## Results

The tropical climate allows for a high mosquito density. Villages and surrounding forests are rife with hard-to-find stagnant pools that create accessible and favorable breeding places for mosquitoes. Villagers opt for practices, which have become woven into their tribal culture, that help them cope with the heat, tiring physical labor and poverty. Drinking *jeeluga-kallu* (indigenous alcoholic brew), misusing bed nets, sleeping without clothing, and disregarding malaria symptoms allow malaria to flourish. The general mistrust of government supplied medication, lack of education and heedlessness paid to government efforts exacerbate the problem. Current malaria initiatives include anti-larval operations, anti-adult operations, repellants and bed nets to prevent bites, and weekly surveillance and administration of medicine when needed by trained health workers [3]. While the protocols seem to encompass the entire problem, malaria continues to plague the tribal area. We found numerous communication gaps within malaria programs and between government and private health centers that can adversely affect program implementation. Also, these remote villages lack proper infrastructure and access to education. This coupled with the government’s lack of proper follow-up and continued care in villages creates loopholes for malaria to slip through.

## Conclusions

Our investigative surveys and fieldwork point to three major factors intertwined in the setback of Rampachodavaram’s malaria control: environment, culture, and infrastructure. The tribals’ primitive lifestyle supports an environment with antiquated drainage, disposal systems, architecture and agricultural apparatuses saturated with breeding places. Many of the practices in the tribals’ day-to-day life have created a culture that is an easy target for mosquitoes. Lastly, a flawed infrastructure hinders efforts; irregularity in treatments and lack of follow-ups are common in the tribal area. If malaria is to be effectively combated in Rampachodavaram, it is imperative that more people become dedicated to the cause and strive to educate about precautions and treatment against malaria.
